# The mental health and well-being benefits of exercise during the COVID-19 pandemic: a cross-sectional study of medical students and newly qualified doctors in the UK

**DOI:** 10.1007/s11845-020-02423-z

**Published:** 2020-11-04

**Authors:** Conor Coyle, Hanya Ghazi, Ioannis Georgiou

**Affiliations:** 1 St. George’s University of London, London, UK; 2 Hull-York Medical School, York, UK; 3 Aberdeen University, Aberdeen, UK

**Keywords:** Coronavirus, COVID-19, Mental health, Medical students, Foundation doctors, Prospective study

## Abstract

**Background:**

University students have been uniquely affected by the COVID-19 pandemic. However, there is currently little data upon the measures that medical students and newly qualified doctors have taken to help their mental well-being and mood during the COVID-19 pandemic.

**Aim:**

We aimed to identify the activities respondents found beneficial for their well-being and mental health and recorded a mood score from survey respondents.

**Methods:**

A nationwide study was completed to investigate the mental health impact of the COVID-19 pandemic upon medical students and newly qualified doctors (interim foundation year one). We received 2075 respondents from across the UK.

**Results:**

Physical activity was found to be the most common activity used by the survey respondents to help with their mental well-being (80.1%) (medical students, 83.7%; interim foundation doctors, 72.3%). Participants who stated that exercise helped their well-being had a mean score (SD) of 52.3 (20.7) which was significantly higher (*P* = 0.048) than those who reported that they did not exercise (49.8 (21.1)). Respondents who stated they had used exercise to help with their mental well-being had (on average) a higher mood score than those who did not. This was seen in both the medical student and interim foundation doctor subgroups.

**Discussion:**

Exercise can help to benefit the well-being of medical students and interim foundation doctors. It is hoped that higher education providers and employers recognise the importance of promoting physical activity for the well-being of their students and staff, respectively.

## Introduction

University students have been uniquely affected by the COVID-19 pandemic [1]. However, there is currently little data upon the measures that medical students and newly qualified doctors have taken to help their mental well-being and mood during the COVID-19 pandemic. As we adjust to the impact of COVID-19 upon our way of life, understanding actions that improve and support mental well-being become especially important, in the development of future health policy and mental health support [2, 3].

Our group hypothesised exercise to be the most common activity of survey respondents. We also hypothesised that survey respondents who exercised would report higher mood scores.

## Methods

A nationwide study was completed to investigate the mental health impact of the COVID-19 pandemic upon medical students and newly qualified doctors (interim foundation year one).

We received 2075 respondents from across the UK (aged 18–59); this included 1909 medical students (92.0%) and 166 newly qualified doctors (0.08%). We aimed to identify the activities respondents found beneficial for their well-being and mental health and recorded a mood score from survey respondents. Respondents were asked to provide a score from 0 to 100 of their mood (0 being the worst and 100 being the best mood they could imagine). SPSS IBM v25 was utilised for the analysis of these results.

## Results

The overall mean mood score of respondents was 51.8, SD 21.1. Physical activity was found to be the most common activity used by the survey respondents to help with their mental well-being 80.1% (medical students 83.7%; interim foundation doctors 72.3%). Participants who stated that exercise helped their well-being had a mean score (SD) of 52.3 (20.7) which was significantly higher *P* = 0.048 than those who reported that they did not exercise, 49.8 (21.1). One-way ANOVA analysis revealed that there was a statistically significant difference (*P* = 0.037) between the mean mood scores (SD) of the following groups: students who did not exercise 49.7 (21.2), students who exercised 52.0 (21.0), doctors who exercised 56.2 (22.7) and doctors who did not exercise 50.9 (19.1).

Scheffe’s post hoc analysis revealed that the statistically significant difference mentioned above was a result of the mean difference between students who did not exercise compared to doctors who exercised, who scored on average 6.5 points lower on mean mood scores.

## Discussion

Respondents who stated they had used exercise to help with their mental well-being had (on average) a higher mood score than those who did not. This can be seen in both the medical student and interim foundation doctor subgroups. These results further demonstrate the benefits of physical activity upon well-being and provide a promising insight into the measures taken by medical students and interim foundation doctors in the UK, to help their mental well-being.

The promotion of exercise during the COVID-19 pandemic has become an objective of governments across the world. The health benefits of exercise and being physically fit have been widely supported as a measure to improve public health against the virus. Moreover, physical activity provides a means to manage negative emotions, positively impacting an individual’s mental health and well-being [4, 5].

As COVID-19 continues to spread globally, medical students and interim foundation doctors will continue to face significant challenges and uncertainty surrounding their education, future careers and wider life. It is hoped that medical students and interim foundation doctors who do not exercise, engage in physical activity—recognising the benefits that physical activity can have for their health and well-being. It is also hoped that higher education providers and employers recognise the importance of promoting physical activity for the well-being of their students and staff, respectively.
